# Diagnosis of orthopaedic-implant-associated infections caused by slow-growing Gram-positive anaerobic bacteria – a clinical perspective

**DOI:** 10.5194/jbji-6-367-2021

**Published:** 2021-10-07

**Authors:** Diana Salomi Ponraj, Thomas Falstie-Jensen, Nis Pedersen Jørgensen, Christen Ravn, Holger Brüggemann, Jeppe Lange

**Affiliations:** 1 Department of Clinical Medicine, Aarhus University, Aarhus, 8000, Denmark; 2 Department of Orthopaedic Surgery, Aarhus University Hospital, Aarhus, 8200, Denmark; 3 Department of Infectious Diseases, Aarhus University Hospital, Aarhus, 8200, Denmark; 4 Department of Orthopaedic Surgery, Lillebaelt Hospital, Kolding, 6000, Denmark; 5 Department of Biomedicine, Aarhus University, Aarhus, 8000, Denmark; 6 Department of Orthopaedic Surgery, Horsens Regional Hospital, Horsens, 8700, Denmark

## Abstract

Slow-growing Gram-positive anaerobic bacteria (SGAB) such as *Cutibacterium acnes* are increasingly recognized as causative agents of implant-associated infections (IAIs) in orthopaedic surgeries. SGAB IAIs are difficult to diagnose because of their non-specific clinical and laboratory findings as well as the fastidious growth conditions required by these bacteria. A high degree of clinical suspicion and awareness of the various available diagnostic methods is therefore important. This review gives an overview of the current knowledge regarding SGAB IAI, providing details about clinical features and available diagnostic methodologies. In recent years, new methods for the diagnosis of IAI were developed, but there is limited knowledge about their usefulness in SGAB IAI. Further studies are required to determine the ideal diagnostic methodology to identify these infections so that they are not overlooked and mistakenly classified as aseptic failure.

## 1 Introduction

Implant-associated infections (IAIs) in orthopaedic surgery, which includes prosthetic joint infections (PJIs) in this review, are associated with high morbidity and mortality (Tande and Patel, 2014; Fischbacher and Borens, 2019) and remain a highly strenuous scenario to encounter for most orthopaedic surgeons and patients alike. Aerobic bacteria, primarily staphylococcal species, are the most identified organisms in culture-positive IAI. Slow-growing Gram-positive anaerobic bacteria (SGAB) are increasingly being recognized as potential agents in IAI, although their role is not well established, and their diagnosis is challenging (Tande and Patel, 2014; Shah et al., 2015; Bjerke-Kroll et al., 2014; Singh et al., 2012; Achermann et al., 2014; Aubin et al., 2014; Portillo et al., 2013; Lin et al., 2020). The purpose of this narrative review is to introduce and highlight all SGAB, including the rarer ones; to evaluate current diagnostic SGAB-IAI information from a clinical perspective, helping to guide the diagnostic process; and to identify areas for future research.

### SGAB-IAI case report

A 68-year-old man presented with complaints of persistent pain from a primary total hip arthroplasty (THA), which had been performed due to idiopathic osteoarthrosis 3 years prior. The immediate post-operative period had been uneventful, but during the following 2 years he experienced increasing hip pain. Due to a medical evaluation of general malaise and chronic elevated serum erythrocyte sedimentation rate (ESR), an ${}^{18}$F-FDG PET/CT (${}^{18}$F-fluorodeoxyglucose positron emission tomography/computed tomography) was performed, which showed increased tracer uptake around the THA, and he was referred to the corresponding author's hip clinic. He had elevated ESR and mildly raised serum C-reactive protein (CRP). Ultrasound-guided hip joint aspiration was performed, and analysis showed a total white blood cell (WBC) count of 55 540 cells per µL and polymorphonuclear neutrophils (PMNs) of 52 170 cells per µL. However, no growth was found in culture after 14 d of incubation on *two* consecutive ultrasound-guided hip joint aspirations.

In view of the patient's symptoms, imaging, and laboratory findings, prosthetic joint infection (PJI) was suspected based on modified Musculoskeletal Infection Society (MSIS) criteria (Falstie-Jensen et al., 2019a), and revision of the THA was planned as per the CORIHA protocol (Lange et al., 2018). By the time the patient was admitted for revision surgery, he had developed a sinus tract from the THA to the skin, which in all classifications is a definite sign of infection. During the one-stage revision, five tissue biopsies were obtained as recommended by Kamme and Lindberg (1981). No growth was seen in any of the cultures after 14 d of standardized incubation.

Sonication of the implant was performed as part of an unrelated research project. Finally, *Cutibacterium acnes* (*C. acnes*) was isolated from the sonicated fluid after 6 d of anaerobic incubation, with no growth presented in the aerobic cultures. The patient was treated as per local hospital guidelines for management of PJI. The patient had no pain or other sign of PJI recurrence at latest follow-up and had an eventless, contralateral, THA in the follow-up period due to idiopathic osteoarthrosis. The patient has given full written consent for the use of his case.

## 2 Slow-growing Gram-positive anaerobic bacteria: definition and species

SGAB comprise Gram-positive bacteria of the two phyla Firmicutes and Actinobacteria that are usually slow-growing and require long cultivation times (>3 d but often longer) when isolated from clinical samples. Their growth relies on anaerobic metabolism with limited or no growth under aerobic conditions. SGAB species that have been reported as causative agents of IAI are listed below.

*Cutibacterium* species (previously *Propionibacterium*): these Gram-positive, aerotolerant, anaerobic coryneform bacteria are part of the normal skin microbiota. The most common species associated with IAI are *C. acnes* (Lutz et al., 2012; Bossard et al., 2016) followed by *C. avidum* (Renz et al., 2018a).

*C. acnes*: although reported as a causative agent of IAI since the 1970s, it has gained more interest in recent years as a biofilm-producing pathogen (Kamme and Lindberg, 1981; Achermann et al., 2014). It is the most common anaerobic bacterium isolated in IAI and is most commonly seen in late onset, chronic PJI (Lebowitz et al., 2017; Triffault-Fillit et al., 2019). It has a predilection for the upper limb and the spine and is considered to be the most common organism causing shoulder PJI (Namdari et al., 2019; Sevelda and Fink, 2018; Achermann et al., 2013; Zhang et al., 2015; Marmor et al., 2016; Kerneis et al., 2017), but it is also found in PJI of hip, knee, and metacarpophalangeal joints, as well as spinal IAI (Drago et al., 2017; Nodzo et al., 2016; Bacle et al., 2017; Garg et al., 2015; Sampedro et al., 2009). It is also the most common bacterium grown in unexpected positive cultures in revision shoulder arthroplasty and removed clavicle plates (Padegimas et al., 2017; Foruria et al., 2013; Lucas et al., 2016; Both et al., 2018; Gausden et al., 2017). *C. acnes* is a normal skin commensal. In patients undergoing primary shoulder surgery, it was found that *Cutibacterium* species were cultured from unprepared epidermal skin surfaces and freshly incised dermal edges in 72 % and 34 %, respectively (Matsen et al., 2020). Studies have also reported the presence of *Cutibacterium* species in deep tissue cultures in patients undergoing primary shoulder arthroplasty despite thorough skin preparation and preoperative intravenous antibiotic prophylaxis (Matsen et al., 2015; Hudek et al., 2014).

*C. avidum*: it is the second most common *Cutibacterium* species associated with IAI (Zeller et al., 2007, 2018; Wildeman et al., 2016; Renz et al., 2018a). It is mostly seen in PJI of the hip, especially when the anterior approach is used, but infections involving fixation plates of the femur, humerus, and prosthetic shoulder joints have also been reported (Renz et al., 2018a; Zeller et al., 2018; Sampedro et al., 2009; Achermann et al., 2017; Boni et al., 2018).

*C. granulosum*: the first case of PJI due to this bacterium was described in 2013 in a patient after THA (Nystrom et al., 2013; Zeller et al., 2018).

*Finegoldia magna* (previously *Peptostreptococcus magnus*): this Gram-positive anaerobic coccus is part of the commensal flora, found on healthy human skin and as a part of the gut microbiota (Söderquist et al., 2017; Levy et al., 2009). Although less frequent than *Cutibacterium* species, PJIs of hip, shoulder, knee, and foot, as well as osteosynthesis-related IAI due to *F. magna*, have been reported (Levy et al., 2009; Söderquist et al., 2017; Renz et al., 2018a; Achermann et al., 2013; Walter et al., 2014; Richards et al., 2014; Kamme et al., 1974; Puchner et al., 2017; Akgun et al., 2020; Rieber et al., 2020; Anagnostakos et al., 2021).

*Staphylococcus saccharolyticus* (previously *Peptococcus saccharolyticus*): this anaerobic coagulase-negative staphylococcus has been isolated from a limited number of cases of shoulder and hip PJI and spinal IAI (Trojani et al., 2020; Evans et al., 1978; Pumberger et al., 2019; Brüggemann et al., 2019; Söderquist et al., 2021).

*Parvimonas micra* (previously *Micromonas micros* or *Peptostreptococcus micros*): a few cases of IAI due to this Gram-positive anaerobic coccus have been reported to date (Renz et al., 2018a; Rieber et al., 2018, 2020; Bartz et al., 2005; Stoll et al., 1996; Z. Huang et al., 2019; Anagnostakos et al., 2021).

*Miscellaneous SGAB*: in addition to the above, IAI due to rare SGAB like *Slackia exigua*, *Robinsoniella peoriensis* and *Facklamia hominis* have also been reported in case reports (Rieber et al., 2016, 2019; Corona et al., 2014). With recent focus on *Cutibacterium acnes* and its role in IAI, many studies have been done on that species. But there is limited information on rarer SGAB, even though their role in IAI is not of any less importance.

## 3 The diagnosis of SGAB IAI

### 3.1 Classification

Regarding the definition of PJI, different classifications have been proposed by the Musculoskeletal Infection Society (MSIS), the Infectious Diseases Society of America (IDSA), and the European Bone and Joint Infection Society (EBJIS) (Parvizi et al., 2011, 2018; Osmon et al., 2013; Renz et al., 2018b; McNally et al., 2021). Although there are differences, several criteria are present throughout all the guidelines, such as the presence of a sinus tract, histopathologic evidence of inflammation, and positive cultures from tissue biopsies or aspirations. In addition, evaluation of inflammatory cells and biomarkers, both in serum and synovial fluid, are also taken into consideration (Renz et al., 2018b; Parvizi et al., 2011, 2018; Osmon et al., 2013). Whether these criteria are stringently applicable for SGAB PJI remains uncertain, amongst others due to these organisms' specific growth requirements and in vivo reduced inflammatory response. Moreover, there are no established or widely accepted diagnostic criteria for non-PJI IAIs, making their diagnosis difficult and limiting the available data on non-PJI IAI, irrespective of the causative organism. As such, no clear classification has been established in regard to SGAB IAI.

### 3.2 Clinical features

Differentiation between SGAB IAI and aseptic failure based only on clinical symptoms is often extremely difficult, and a high degree of clinical suspicion is warranted, with pain being the most predominant feature.

Persistent or increasing pain from an area with an orthopaedic implant and joint stiffness in specific relation to PJI are the most common clinical symptoms reported in SGAB IAI. However, such symptoms are also frequently present in cases of aseptic failure (Renz et al., 2018a; Rienmuller and Borens, 2016; Jacobs et al., 2016; Wang et al., 2013; Hsu et al., 2018). The classical clinical features of IAI include fever, local signs of inflammation such as redness or warmth, pain and swelling, and a sinus tract. Fever and local signs of inflammation are not just non-specific but also observed less frequently in SGAB IAI than pain and joint stiffness (Rieber et al., 2016; Rieber et al., 2019; Corona et al., 2014; Maroto Piñeiro et al., 2021; Randall et al., 2020; Z. Huang et al., 2019). The frequency of a sinus tract in SGAB IAI has been reported anywhere from less than 1 % up to 20 % (Renz et al., 2018a; Rienmuller and Borens, 2016; Dodson et al., 2010; Jacobs et al., 2016).

Several groups have analysed the clinical significance of unexpected positive cultures (UPCs) of *C. acnes* in revision shoulder surgery and found that they had no clinical relevance in at least 25 % of UPC cases (Foruria et al., 2013; Falstie-Jensen et al., 2021).

### 3.3 Medical imaging

Medical imaging is applied as routine first-line investigation in patients with orthopaedic implants. But plain radiographs are neither sensitive nor specific for the diagnosis of IAI, irrespective of the causative organism as it is difficult to differentiate between infection and aseptic loosening based on a single X-ray (Gemmel et al., 2012).

Only a few studies have distinctly described X-ray findings in SGAB IAI, with signs of loosening being the most frequent finding seen in 23 %–73 % of *C. acnes* IAI as well as in IAI due to less frequent SGAB (Renz et al., 2018a; Rienmuller and Borens, 2016; Wang et al., 2013; Hou et al., 2015; Pottinger et al., 2012; Rieber et al., 2016, 2019; Corona et al., 2014). However, it is difficult, if not impossible, to differentiate between aseptic and septic loosening (Lima et al., 2013) in these cases. Signs of osteitis, with osteolysis and/or periosteal bone formation, or abscess formation are some of the reported X-rays and ultrasonographic findings, respectively, of *C. avidum*-associated IAI (Zeller et al., 2018; Wildeman et al., 2016). Nuclear medicine imaging has not been found useful in the diagnosis of SGAB in chronic shoulder PJI (Falstie-Jensen et al., 2019b, c).

### 3.4 Serum and synovial biomarkers

In general, SGAB do not elicit a strong inflammatory response. Consequently, conventional inflammatory markers may not be elevated (Sampedro et al., 2009), and as such the value of existing serum and synovial biomarkers in SGAB IAI is questionable.

Several studies indicate that the WBC count is in the normal range in SGAB IAI (Renz et al., 2018a; Zappe et al., 2008; Sampedro et al., 2009; Nodzo et al., 2016) and that this also occurs in IAI due to more virulent organisms (Nodzo et al., 2016; Akgun et al., 2019). While ESR and CRP perform better compared to WBC count in the diagnosis of *C. acnes* IAI (Dodson et al., 2010), ESR/CRP cannot clearly differentiate between aseptic failure and SGAB IAI (Sampedro et al., 2009; Plaass et al., 2016). They are not always elevated in *C. acnes* IAI (Renz et al., 2018a; Figa et al., 2017), and when elevated, their levels are still lower compared to IAI caused by highly virulent microorganisms (Akgun et al., 2018; Grosso et al., 2014a; Nodzo et al., 2016). Normal ESR and CRP have also been reported in IAI caused by infrequent SGAB (Z. Huang et al., 2019; Corona et al., 2014). It should be noted that comparison between different studies is difficult, as different cut-off values were used for CRP (>5 mg L${}^{-1}$; Plaass et al., 2016; Zeller et al., 2018; >7 mg L${}^{-1}$; Dodson et al., 2010, or >10 mg L${}^{-1}$) (Renz et al., 2018a; Nodzo et al., 2016; Grosso et al., 2014a; Akgun et al., 2018; Figa et al., 2017), while the latest EBJIS definition of PJI mentions that a CRP > 10 mg L${}^{-1}$ is suggestive of “likely infection” (McNally et al., 2021).

Synovial fluid and serum levels of interleukin-6 (IL-6) have been identified with a high sensitivity and specificity in the diagnosis of PJI of hip and knee in general, but little is known about their usefulness in SGAB IAI (Deirmengian et al., 2010; Alijanipour et al., 2013). Serum IL-6 has potentially a poor sensitivity and specificity and cannot currently be recommended as a preoperative diagnostic test in SGAB IAI (Frangiamore et al., 2015; Grosso et al., 2014b).

Synovial alpha defensins (α-defensin), an antimicrobial peptide, can be measured by enzyme-linked immunosorbent assay (ELISA) or by α-defensin lateral flow (ADLF) tests and has been included in the recent EBJIS definition of PJI (Weigelt et al., 2020; McNally et al., 2021). In the diagnosis of *C. acnes* shoulder PJI, studies have shown that synovial α-defensins by ELISA (Unter Ecker et al., 2019; Frangiamore et al., 2015) may have a role to play in *C. acnes* IAI, but Weigelt et al. (2020) found that the ADLF test failed to identify the only culture-positive *C. acnes* shoulder PJI in their study (Weigelt et al., 2020). Overall, it is difficult to assess the ADLF test's potential in SGAB IAI of hip and knee, as few or no SGAB were included in any of the studies that showed high sensitivity and specificity of the ADLF in hip and knee PJI (Deirmengian et al., 2014; Bonanzinga et al., 2017; Gehrke et al., 2018). Focus of future studies should be on evaluating the ELISA test in SGAB IAI.

The simple and inexpensive leukocyte esterase strip test (also used for urinary tract infections) has an overall concordance of 86 %–93 % with culture results in hip and knee PJI in general (Di Benedetto et al., 2019). But its use in SGAB IAI might be limited, as the test could only correctly identify 3 of 15 *C. acnes* shoulder PJIs (Unter Ecker et al., 2019).

Other biomarkers like serum D-dimer and synovial CRP are also included as minor criteria in an updated PJI definition (Parvizi et al., 2018). However, studies on D-dimer have shown conflicting results in IAI in general (Shahi et al., 2017; J. Huang et al., 2019), similarly contradictive results have been seen in the role of synovial CRP (Omar et al., 2015; Tetreault et al., 2014), and their use in SGAB IAI has not been evaluated. Serum procalcitonin has also previously been evaluated and not found to be of value in the diagnosis of PJI in general (Xie et al., 2017; Saleh et al., 2018).

### 3.5 Synovial fluid cytology

Aspiration of synovial fluid is often performed in suspected cases of PJI. When no lavage has been performed and clear fluid is aspirated, synovial fluid cytology gives valuable information about the level of local inflammation (McNally et al., 2021). Elevated synovial WBC count is regarded as a diagnostic criterion in all major definitions of PJI (McNally et al., 2021; Parvizi et al., 2018; Osmon et al., 2013). But different studies have mentioned different cut-offs. The latest EBJIS definition suggests synovial fluid WBC count of >3000 cells/µL and the presence of >80 % PMNs as likely cut-offs for PJI of hip and knee (McNally et al., 2021). These values are not applicable in PJI of upper limbs and can also be affected by use of immunosuppressive drugs or presence of co-morbid conditions like inflammatory arthritis, metal-on-metal prosthesis, or crystal-induced arthritis (Ottink et al., 2019).

However, synovial fluid WBC count is not always increased in SGAB IAI, and when elevated, their numbers are similar to that seen in more virulent microorganisms (Renz et al., 2018a; Nodzo et al., 2016). In *C. avidum* PJI, the median synovial WBC count was reported to be high with a median differential count of 95 % PMNs (Zeller et al., 2018).

### 3.6 Histopathology

Histopathological examination revealing the presence of neutrophils is an important criterion in the overall diagnosis of PJI. In the 2021 EBJIS definition of PJI, the presence of ≥5 neutrophils in ≥5 high-power field (HPF or 400× magnification) or presence of ≥5 neutrophils in a single HPF indicate confirmed or likely infection, respectively (McNally et al., 2021). However, there is a difference in histopathologic findings in IAI caused by *C. acnes* and *C. avidum*, with *C. avidum* causing a more robust immune response. Renz et al. (2018a) reported that inflammation was seen in peri-implant tissue in 100 % of IAI caused by *C. avidum*, but when cases of *C. acnes* were included in the evaluation, the overall positivity dropped to 67 % (Renz et al., 2018a). Histopathological examinations of intraoperative peri-implant tissue samples are frequently negative for the presence of neutrophils, with a positivity of only about 17 %–50 % in *C. acnes*-associated PJI (Figa et al., 2017; Sampedro et al., 2009; Butler-Wu et al., 2011; Grosso et al., 2014a).

Interestingly, Hudek et al. (2021) used immunohistochemistry with *C. acnes*-specific antibodies on tissue samples taken from patients undergoing primary shoulder surgery or arthroscopy for the first time and detected *C. acnes* intracellularly within stromal cells and macrophages (Hudek et al., 2021). While the results of their study point towards *C. acnes* being a commensal in the shoulder, immunohistochemistry can also be potentially used to diagnose SGAB IAI with histopathology.

### 3.7 Microbiology

Microbiology remains the most important clinical entity in IAI as this, besides confirming the diagnosis (McNally et al., 2021), also provides a potential antibiogram to help guide the clinician in the adjuvant antibiotic treatment following surgery. The methods can be culture dependent or culture independent. However, especially concerning SGAB IAI there, are many aspects to take into consideration for the clinician, as SGAB by nature demands sophisticated processing not always undertaken in an everyday clinical setting, unless the clinician is acutely aware of the potential presence of SGAB as the causative organism.

#### 3.7.1 Culture-dependent methods

Culture-dependent methods are relatively cheap and readily available in any clinical microbiology laboratory. The success of culture-dependent methods depends on several factors, e.g. the nature of the organism to be isolated, the quality, type and number of specimens collected, and conditions and duration of incubation (Larsen et al., 2012). Samples for microbiological diagnosis of IAI can be obtained preoperatively or intraoperative, as aspirations or biopsies, but also following implant subjected to sonication (Zimmerli et al., 2004). In case of culture-negative PJI, culture-independent methods, such as next-generation sequencing (NGS), might be a useful addition to identify the causative SGAB (Tarabichi et al., 2018).

Aspiration appears most useful in *C. avidum* IAI with one study showing the growth of this bacterium in preoperative aspiration fluid in 14 out of 15 cases (Zeller et al., 2018). It has been found that *C. acnes* IAI culture of synovial/peri-implant fluid was not diagnostically accurate and showed low sensitivity compared to sonication fluid and peri-implant tissue (Renz et al., 2018a; Rienmuller and Borens, 2016; Figa et al., 2017; Dilisio et al., 2014). As in our case, culture-negative aspirations based on standard culture methods, with clear indications of infections based on synovial biomarkers, could indicate the presence of SGAB as the causative organism.

Culture of tissue samples is not just one of the oldest but also one of the most important methods in the diagnosis of IAI (Kamme et al., 1974; Kamme and Lindberg, 1981; Osmon et al., 2013; Parvizi et al., 2018). The method of collection and the number of samples collected can affect the culture results. To optimize the chance of culturing bacteria, antibiotic pause 2 weeks preoperatively and prophylactic antibiotics withheld until the samples are collected are often applied, although the value in SGAB IAI has not been investigated (Atkins et al., 1998). Multiple samples, also more than normal, may be required in SGAB due to a combination of low bacterial burden and uneven distribution of bacteria in the peri-implant tissues, as well as the risk of contamination in the processing of the samples (Atkins et al., 1998; McGoldrick et al., 2015). The current IDSA guidelines recommend at least three and optimally five or six samples with two positive cultures for a definitive diagnosis (Kamme and Lindberg, 1981; Osmon et al., 2013). However, a larger number of samples may be required for SGAB which have a lower rate of positive culture (Nodzo et al., 2016; McGoldrick et al., 2015; Akgun et al., 2018; Kheir et al., 2018). Kheir et al. (2018) calculated that to get two positive cultures the average number of samples required would be 10 for *C. acnes*, while *S. aureus* and coagulase-negative staphylococci (CoNS) would need just 3 and 4 samples, respectively (Kheir et al., 2018). But with the increase in number of samples comes a theoretic increase in false positive cultures due to contamination. The specific aspects in relation to SGAB IAI need further evaluation.

Since the introduction of sonication of removed implants in the 1990s by Tunney et al. (1998) and further improvements by Trampuz et al. (2007), microbial detection in sonication fluid culture has become widely adopted. Sonication is now incorporated in the IDSA and EBJIS guidelines for diagnosis of PJI (Osmon et al., 2013; McNally et al., 2021). Like other IAI-associated bacteria, SGAB have been shown to form biofilms on medical devices and sonication helps to dislodge these biofilms, making it easier to culture the bacteria (Achermann et al., 2014; Trampuz et al., 2007). Moreover, in patients with prior antibiotic therapy, sonication fluid specimens have shown to give more sensitive culture results than tissue specimens (Trampuz et al., 2007). For IAI caused by anaerobes, the culture of sonication fluid appears more sensitive than periprosthetic tissue samples (Portillo et al., 2014). Yet, several studies could not find any advantage of sonication fluid over tissue specimens in *C. acnes* IAI (Renz et al., 2018a; Akgun et al., 2020; Grosso et al., 2018). Conversely, the use of sonication fluid specimens was found to be inferior to the use of peri-implant tissue specimens in the detection of *C. acnes* in spinal implant infections (Sampedro et al., 2010).

The results from available studies regarding SGAB cultures are not easily comparable, as results depend on various factors, such as method of sample processing, choice of culture media, duration of incubation, and the cut-off chosen to determine significant microbial growth. For the processing of tissue samples, different methods of homogenization have been mentioned (Renz et al., 2018a; Figa et al., 2017; Zeller et al., 2018; Kheir et al., 2018; Grosso et al., 2018), and these could possibly affect the culture results too. Culture media used for the growth of anaerobic bacteria vary among studies. Anaerobic sheep blood agar (Renz et al., 2018a; Baumbach et al., 2018; Akgun et al., 2020; Grosso et al., 2018) and Columbia blood agar (Dilisio et al., 2014; Zeller et al., 2018) were the most commonly used solid media, while thioglycolate broth (19, 33, 89, 92, 93) was the most commonly used liquid media. In addition, automated culture systems, like BACTEC or BacT/Alert, which are routinely used for blood culture and usually have the advantage of shorter time to positivity compared to conventional methods, were also used in some studies (Renz et al., 2018a; Portillo et al., 2014; Minassian et al., 2014; Sanabria et al., 2019). However, no consensus on the choice of media for SGAB exists, which makes evaluation of SGAB IAI a difficult task, as these IAIs can be potentially in an everyday clinical setting. The duration of cultivation is another important factor to consider, when it comes to detection of SGAB. Prolonged incubation of SGAB is usually required compared to other organisms. Previous studies have shown that while most bacteria causing orthopaedic infections were isolated within 1 to 5 d, *C. acnes* can take anywhere from 6 to 14 d or longer to grow (Kheir et al., 2018; Kerneis et al., 2017; Nodzo et al., 2016; Garg et al., 2015). Incubation for 14 d seems to be the current standard for SGAB IAI (Renz et al., 2018a; Figa et al., 2017; Zeller et al., 2018; Baumbach et al., 2018; Nodzo et al., 2016; Kheir et al., 2018; Akgun et al., 2020; Portillo et al., 2014), though other studies have used 21 d (McGoldrick et al., 2015; Dilisio et al., 2014) and even 28 d (Lucas et al., 2016; Matsen et al., 2013). The need for prolonged incubation times must be considered against the increased risk of detection of contaminants with longer incubation periods. The number of bacterial colonies (colony forming units, CFU) that is cultivated is an important criterion to distinguish a true infection from possible contamination, and this topic is not evaluated for SGAB IAI. In sonication fluid different diagnostic CFU cut-offs (20, 50 CFU mL${}^{-1}$, etc.) have been applied (Akgun et al., 2020; Grosso et al., 2018). The latest EBJIS definition of PJI (McNally et al., 2021) set the cut-off for confirmed infection to >50 and >200 CFU mL${}^{-1}$ of any organism for uncentrifuged and centrifuged sonication fluid, respectively. Infection with SGAB was not differentiated in these guidelines (McNally et al., 2021).

#### 3.7.2 Culture-independent methods

Culture-independent methods may have an important role in diagnosis of IAI, when other diagnostic tests are inconclusive, e.g. in the case of fastidious, slow-growing, or viable but non-culturable bacteria, and in patients with prior antibiotic therapy (McNally et al., 2021; Parvizi et al., 2018; Pasquaroli et al., 2013). Commonly used culture-independent methods include polymerase chain reaction (PCR)-based methods such as species-specific PCR, multiplex PCR, and 16S ribosomal RNA (rRNA) PCR and next-generation sequencing. The clinical downside to culture-independent methods is the lack of an antibiogram in the individual case.

The usefulness of PCR assays in IAI is debated. Sampedro et al. (2010) developed a species-specific *C. acnes* PCR assay, which amplifies a region of the *C. acnes* 16S rRNA gene. The application of this *C. acnes* PCR assay to sonication fluid was compared with the results from sonication fluid culture. The PCR assay was able to detect only six of the nine *C. acnes*-positive cultures (Sampedro et al., 2010). The use of multiplex PCR assays that allow the simultaneous detection of multiple species of choice have yielded conflicting results. Morgenstern et al. (2018) used multiplex PCR and found that synovial fluid multiplex PCR was superior to culture for the detection of hip and knee PJI due to low virulent organisms (not SGAB) and gave earlier results (5 h versus up to 14 d) (Morgenstern et al., 2018). However, other studies found that multiplex PCR of sonication fluid detected fewer pathogens compared to periprosthetic tissue culture and sonication fluid culture and missed several low virulent organisms, including SGAB (*Cutibacterium* spp. and *Finegoldia magna*) (Renz et al., 2017, 2018c). Bemer et al. (2014) found that the sensitivity of 16S rRNA gene PCR assay was low for *C. acnes*, with only 11 % of *C. acnes* and 72 % of CoNS infections being detected by PCR, compared with 92 % and 100 % of *S. aureus* and streptococci, respectively (Bemer et al., 2014). The role of PCR based methods in SGAB IAI needs further evaluation. Amplicon next-generation sequencing (aNGS) or metagenomic next-generation sequencing (mNGS) have not been used, clinically, in the past for the diagnosis of IAI but is increasingly used in research on IAI. Its applicability is currently limited due to high costs and the need for extensive bioinformatics analyses but may have a future genuine clinical value in SGAB IAI. It involves DNA or RNA extraction, PCR amplification of target region (aNGS), enrichment of bacterial DNA or RNA (mNGS), library preparation, sequencing, and bioinformatics analysis (Chiu and Miller, 2019). The analysis can be targeted in aNGS, which involves the amplification of specific genomic regions of believed value or untargeted in mNGS (Chiu and Miller, 2019). The details and protocols of the individual steps can vary between individual laboratories, as these are currently at an experimental level. Next-generation sequencing analysis can be performed on any sample that yields sufficient nucleic acid (Chiu and Miller, 2019). Wang et al. (2020a) found in a study of 24 culture-negative PJIs that mNGS was able to detect rare pathogens and fastidious bacteria like *Mycoplasma hominis*, *F. magna*, and *P. micra*, which were clinically believed to be the causative organisms (Wang et al., 2020a). Wang et al. (2020b) found that the sensitivity of mNGS was significantly higher than the sensitivity of standard culture method, but not different to PCR assays (Wang et al., 2020b). Weaver et al. (2019) found in joint fluid from seven PJI cases that mNGS was able to detect more species including *C. acnes* and also was superior for the detection of polymicrobial infections compared to standard culture (Weaver et al., 2019). Namdari et al. (2019) found poor correlation between mNGS and culture in the detection of shoulder PJI (Namdari et al., 2019).

## 4 Limitations of the review

Most studies mentioned in this review investigated the diagnostic challenges and frequencies of PJI due to *Cutibacterium* spp. SGAB other than *Cutibacterium* species present with similar diagnostic challenges because of their similar clinical, imaging, laboratory, and microbiological findings, but only a limited number of studies focus on these SGAB in particular.

Another limitation of the review is the underrepresentation of non-PJI IAI, since only a few studies focus on non-PJI IAI. This could be due to a lack of definitive diagnostic criteria for non-PJI IAI. But SGAB have been reported from IAI other than PJI, and their role in these infections should be investigated in more detail in future studies.

## 5 Conclusion

The presented case and review are in our opinion a clear example of the difficulties that SGAB IAIs present to orthopaedic surgeons, infectious disease specialists, and clinical microbiologists in performing best-practice of care for patients. Currently, a high degree of clinical suspicion based on up-to-date knowledge of SGAB is the only factor that can guide the clinician. Future research into diagnosing SGAB IAI, as specified above, is unquestionably warranted.

## Data Availability

No data sets were used in this article.
